# Nano-layered surface plasmon resonance-based highly sensitive biosensor for virus detection: A theoretical approach to detect SARS-CoV-2

**DOI:** 10.1063/5.0046574

**Published:** 2021-06-14

**Authors:** Md. Moznuzzaman, Imran Khan, Md. Rafiqul Islam

**Affiliations:** 1 Department of Electrical and Electronic Engineering, Jashore University of Science and Technology, Jashore 7408, Bangladesh; 2 Department of Electrical and Electronic Engineering, Khulna University of Engineering and Technology, Khulna 9203, Bangladesh

## Abstract

The outbreak of the coronavirus disease (COVID-19) pandemic has become a worldwide health catastrophe instigated by Severe Acute Respiratory Syndrome Coronavirus-2 (SARS-CoV-2). Countries are battling to slow the spread of this virus by testing and treating patients, along with other measures such as prohibiting large gatherings, maintaining social distance, and frequent, thorough hand washing, as no vaccines or medicines are available that could effectively treat infected people for different types of SARS-CoV-2 variants. However, the testing procedure to detect this virus is lengthy. This study proposes a surface plasmon resonance-based biosensor for fast detection of SARS-CoV-2. The sensor employs a multilayered configuration consisting of TiO_2_–Ag–MoSe_2_ graphene with a BK7 prism. Antigen–antibody interaction was considered the principle for this virus detection. Immobilized CR3022 antibody molecules for detecting SARS-CoV-2 antigens (S-glycoprotein) are used for this sensor. It was found that the proposed sensor’s sensitivity (194°/RIU), quality factor (54.0390 RIU^−1^), and detection accuracy (0.2702) outperformed those of other single and multilayered structures. This study could be used as a theoretical base and primary step in constructing an actual sensor.

## I. INTRODUCTION

Severe Acute Respiratory Syndrome Coronavirus-2 (SARS-CoV-2), a positive-sense single-stranded RNA (ribonucleic acid) virus, has been the cause for the coronavirus disease (COVID-19) pandemic due to its extremely contagious nature. At present, no specific medicine or vaccine has been developed that could fight against all the variants of this virus in humans. Fortunately, infected people can be detected through advanced medical diagnostics.

Nowadays, this diagnosis is conducted through the real-time reverse transcription-polymerase chain reaction (RT-PCR) test. Typically, a sample of mucus is collected from the suspected person’s nose or throat. In RT-PCR technology, the genetic material of the virus is investigated. The viral genetic material is amplified through this technique, and if the person is infected actively, it can be detected. To date, this is the most reliable test, but it is unable to identify a person who has been infected recently. This is because the virus takes a few days to replicate in the nose or throat. Often, the swabs might fail to pick up indications of active infection (Harris, 2020). Notably, this analysis takes a long time to get results. A rapid RT-PCR test, which takes less than 15 min, does exist (Harris, 2020). However, it was found that this test has a false negative rate of 14.8% (Stein, 2020). “Moreover, virus isolation, serological methods and PCR-based assays often require highly trained lab workers and time-intensive procedures, as well as a highly sterile experimental environment” (Bai *et al.*, 2012). Apart from the RT-PCR test, there are several other types of tests available, as listed in Table I. However, they all have limitations as indicated.

**TABLE I.** Common test types and their limitations for SARS-CoV.

Test type	Limitations	References
Real-time reverse transcription-polymerase chain reaction (RT-PCR)	Statistically, about 50% of SARS patients cannot be recognized at a primary phase depending on the viral RNA recognition;	(Li *et al.*, 2005; Huang *et al.*, 2009; Bai *et al.*, 2012; and Manopo *et al.*, 2005)
	Time consuming process;	
	Equipment is very expensive	
Real-time loop-mediated amplification assay	Lower sensitivity than RT-PCR	Huang *et al.* (2009)
Gold film with an enzymatic electrochemical genosensor	Lower sensitivity than RT-PCR	Abad-valle *et al.* (2005)
Rolling circle amplification PCR-based assay	Lower sensitivity than RT-PCR	Wang *et al.* (2005)
Antigen-capturing enzyme-linked immunosorbent assay (ELISA)	Low sensitivity	Shi *et al.* (2003)
Immunofluorescence assay (IFA)	There is a chance of infection while treating the patient with a live virus	Manopo *et al.* (2005)

Considering the limitations of the available SARS-CoV detection technologies, “a possible approach is to detect specific SARS-CoV antigens such as the spike protein” (Huang *et al.*, 2009). It was found that “The Spike protein may mediate membrane fusion and induce neutralization antibodies in the host, raising the possibility that antibodies against the SARS-CoV Spike protein may be a good marker for early detection and neutralization of SARS-CoV infections” (Manopo *et al.*, 2005). Therefore, detection of the SARS-CoV spike protein could be a rapid, accurate, and highly sensitive diagnostic method. In addition, it eliminates the risk of contamination. The objective of this study is to propose a theoretical design of a surface plasmon resonance (SPR)-based biosensor that could detect SARS-CoV-2 through an easy and fast procedure.

Numerous studies in the literature found the SPR-based biosensor to be an effective, label free, and fast detector for different pathogens, including viruses. Earlier, it was reported that an SPR-based biosensor was able to detect feline calicivirus in 15 min (Bai *et al.*, 2012). A similar finding was also obtained for human enterovirus 71 (EV71) (Prabowo *et al.*, 2017). It was found that using an SPR-based biosensor, EV71 could be detected within several minutes.

This type of sensor was thus chosen for this design due to its remarkably high sensitivity, detection accuracy, biocompatibility, and, most importantly, speedy detection. Due to these characteristics, it is becoming a crucial sensing technology in the field of biology (Ahmed and Shaban, 2020), chemistry (Wang *et al.*, 2017), and engineering (Khan and Rahman, 2016; Khan, 2012). The SPR-based sensor is being used in many different applications (Abdulhalim *et al.*, 2008; Bijalwan and Rastogi, 2018; and Nazem *et al.*, 2020). As the focus of this proposed sensor is virus detection, previous literature that considered SPR-based biosensors for virus detection would be highlighted in brief. The sensing theory of these sensors is widely available in the literature, for example, see Homola (2006).

Many recent studies have considered the SPR-based sensor for medical diagnostic applications. For example, Bai *et al.* (2012) employed DNA (deoxyribonucleic acid) aptamers as a specific identifying component in a transportable SPR-based sensor to detect avian influenza virus (AIV) H5N1. The DNA aptamers showed strong binding affinity and high specificity to target AIV H5N1 (Wang *et al.*, 2013). Thus, Bai *et al.* (2012) used this aptamer as they found that it “show(s) comparable affinity for target viruses and better thermal stability than monoclonal antibodies.” However, this detection took about 1.5 h. In contrast, an antibody-based SPR sensor was used to detect feline calicivirus, a surrogate of norovirus, and took only 15 min (Yakes *et al.*, 2013). Another study used a pair of aptamers (IF10 and IF22) and developed a sandwich-type SPR biosensor and detected H5N1 whole virus from infected feces (Nguyen *et al.*, 2016).

An impetuous identification of avian influenza virus subtype H6 was proposed through optical fiber-based SPR sensors in Zhao *et al.* (2016). Both the core diameter and cladding thickness of 62.5 *µ*m of a graded-index multimode optical fiber were adopted for this sensor. The fiber was side-polished, and a thin gold film of 40 nm thickness was used for the surface plasmon, with a sensing area of 5 mm. In an earlier study, a collection mode SPR sensor was proposed, with some advantages over traditional reflectance based measurement such as a high signal to noise ratio, less dependency on metal film thickness, and detection of seasonal influenza A virus, which were demonstrated to show its applicability (Francois *et al.*, 2011). In another study, to diagnose different stages of infections caused by the Epstein–Barr virus, an SPR-based biosensor was proposed, which was able to detect the virus from a clinical serum sample (Riedel *et al.*, 2014). In this sensor, predominantly, the antibodies were detected against the three antigens of the virus.

A localized SPR characteristic was employed to design a gold nanorod biosensor to detect hepatitis B virus (Wang *et al.*, 2010). In detecting the virus, the surface of the nanorod was changed with physical adsorption of the monoclonal hepatitis B surface antibody (HBsAb). This sensor was able to detect the virus in buffer, blood serum, and plasma. On the other hand, Choi *et al.* (2014) proposed a plasma-treated parylene-N film based SPR biosensor for human hepatitis B virus. It was claimed by the authors that “the SPR biosensor with the plasma-treated parylene-N film could achieve more than 1000-fold improved sensitivity in comparison with the conventional ELISA (enzyme-linked immunosorbent assay) kit” (Choi *et al.*, 2014). Similarly, Uzun *et al.* (2009) used a hepatitis B surface antibody on a gold SPR chip surface to detect HBsAb in human serum. In a similar fashion, an SPR-based biosensor biochip was also developed to detect infectious bursal disease virus (Hu *et al.*, 2012).

Recently, an optical sensor (prism-based) used the SPR method to diagnose the dengue virus (DENV) E-protein (Omar *et al.*, 2018). The dengue virus E-protein was detected “by measuring the SPR signal when IgM immobilized gold/Fe-MPA-NCC-CTAB/EDC-NHS thin film is exposed to the DENV E-protein solution” (Omar *et al.*, 2018). For this sensor, the concentration range was varied between 0.0001 and 10 nM.

Apart from these, Shi *et al.* (2015) developed a biosensor to detect nine respiratory viruses: respiratory syncytial virus (RSV), influenza A and B, H1N1, adenovirus, parainfluenza virus 1–3 (PIV1, 2, and 3), and SARS. They employed a gene chip SPR-based biosensor for this detection. In brief, “The respiratory virus target gene was extracted from bacterial culture samples and amplified by PCR technology; PCR products were then analyzed by SPR technology” (Shi *et al.*, 2015). Thus, this virus detection scheme requires a lengthy procedure. Many other previous studies proposed a multilayered (e.g., gold and metamaterials) SPR-based biosensor (Cherifi and Bouhafs, 2017). However, this was not used for virus detection.

Most previous studies used either optical fiber-based (Zhao *et al.*, 2019) or single layered structures for virus detection, using the surface plasmon resonance technique. In contrast, in our proposed sensor, a multilayered structure was used for potential SARS-CoV-2 detection. A single layer structure’s metal film might corrode in bio-solution and would consequently reduce the sensor’s quality factor and sensitivity (Wu *et al.*, 2019); thus, this multi-layer structure was chosen. In addition, recent research found that “the use of graphene and other layered materials for passivation and functionalization broadens the range of metals which can be used for plasmonic biosensing and increases the sensitivity by 3–4 orders of magnitude, as it guarantees stability of a metal in liquid and preserves the plasmonic resonances under biofunctionalization” (Wu *et al.*, 2019).

The rest of this article is organized as follows: Sec. II shows the proposed sensor structure. Section III explains the mathematical modeling and method used for this study. Finally, the results are discussed in Sec. IV before concluding the article.

## II. PROPOSED SENSOR STRUCTURE

**FIG. 1.**
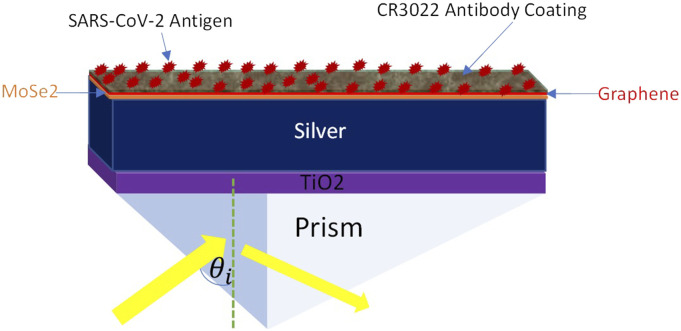
Proposed sensor structure. Thicknesses of different layers are: Graphene: 0.34 nm; MoSe_2_: 0.97 nm; Silver: 58 nm; and TiO_2_: 5 nm.

The proposed sensor structure comprises six layers with different materials, as shown in Fig. 1. Silver is used in this sensor for its appreciable plasmonic behavior in SPR-based sensors. In this configuration, to make a sandwich-like structure, a layer of silver (Ag) is inserted between a MoSe_2_-graphene composite layer and a TiO_2_ thin sheet. The optical nonlinearity improved with higher operating frequency, and overall performance also improved with the smallest Kerr effect at low operating frequency (Maurya *et al.*, 2015a). Therefore, the operating light wavelength is chosen as *λ* = 633 nm, and all the refractive index (RI) values of each material are considered at this wavelength.

In this formation, the most practiced constituent is a prism (BK7 glass) with an RI of 1.5151 (Lu *et al.*, 2012). The subsequent section on the prism is a TiO_2_ layer with an RI of 2.5837 (Maurya *et al.*, 2015a). Previous studies found that the inclusion of oxide layers such as TiO_2_ increases the sensor’s sensitivity. For instance, Singh *et al.* (2013) found that out of three oxide layers, namely, TiO_2_, SiO_2_, and SnO_2_, the maximum sensitivity was obtained for TiO_2_. In addition, TiO_2_ is used as the adhesion layer; this makes the nanostructure mechanically stable. Therefore, they can be integrated with a more complicated device or used as plasmonic sensors for liquid or even solid analytes (Ovchinnikov and Shevchenko, 2013). Furthermore, TiO_2_ has a high light-trapping ability when used as an adherence layer on the prism of an SPR-based sensor. Because of greater light-trapping capability, more surface plasmons (SPs) are generated that eventually increase the resonance angle. This increase in the resonance angle will increase the SPR sensing (Maurya *et al.*, 2015b). Due to these advantages of the TiO_2_ layer, this was employed in this proposed sensor. The succeeding layer is silver with a complex RI of 0.1726 + j3.421 (Verma *et al.*, 2015a). The next two layers are MoSe_2_ and graphene with RIs of 4.7954 + j1.2405 (Beal and Hughes, 1979) and 3.0 + j1.1487 (Maharana *et al.*, 2015), respectively. Finally, to adhere with SARS-CoV-2 testing, the recombinant monoclonal antibody named CR3022 is coated on the graphene layer. The thickness of each layer is also mentioned in Fig. 1.

## III. MATHEMATICAL MODELING AND METHOD

The enumeration of reflected light intensity has been calculated by using the matrix method as this method requires no approximation for multilayer systems (Verma *et al.*, 2015b). *T*_*k*_ is the thickness along the *z*-axis of each layer of the nanocomposite sensor structure. *n*_*k*_ is used to represent the RI, and *ϵ*_*k*_ is used to indicate the permittivity of the material used in the *k*th layer.

The tangential components of both the electric and magnetic fields are continuous at the first surface of the first layer to the last edge of the last layer (Maurya and Prajapati, 2016),

$$\left[\begin{array}{c} U_{1}\\ v_{1} \end{array}\right]=M\left[\begin{array}{c} U_{N-1}\\ v_{N-1} \end{array}\right],$$ (1)

where *U*_1_ is the marginal value of the electric field component and *v*_1_ stands for the marginal component of the incident magnetic field at the margin of the first surface represented in Eq. (1). *U*_*N*−1_ and *v*_*N*−1_ are the corresponding electric and magnetic fields at the border of the *N*th layer, respectively. The characteristic matrix of the composite assembly is represented by *M*_*ij*_, and this equation for *p*-polarized light can be written as follows (Mishra *et al.*, 2016):

$$M_{ij}={\left(\prod_{k=2}^{N-1}M_{k}\right)}_{ij}=\left(\begin{array}{cc} M_{11} & M_{12}\\ M_{21} & M_{22} \end{array}\right),$$ (2)

with

$$M_{ij}=\left(\begin{array}{cc} \cos\;\beta_{k} & -\left(i\;\sin\;\beta_{k}\right)/q_{k}\\ -\left(iq_{k}\;\sin\;\beta_{k}\right) & \cos\;\beta_{k} \end{array}\right),$$ (3)

where

$$q_{k}={\left(\frac{\mu_{k}}{\varepsilon_{k}}\right)}^{1/2}\cos\;\theta_{k}=\frac{{\left(\varepsilon_{k}-n_{1}^{2}\;sin^{2}\;\theta_{1}\right)}^{1/2}}{\varepsilon_{k}}$$ (4)

and

$$\beta_{k}=\frac{2\pi }{\lambda }n_{k}\;\cos\;\theta_{k}\left(Z_{k}-Z_{k-1}\right)=\frac{2\pi T_{k}}{\lambda }{\left(\varepsilon_{k}-n_{1}^{2}\;sin^{2}\;\theta_{1}\right)}^{1/2}.$$ (5)

After some upfront mathematical calculating steps without assumption, the coefficient of reflected light represented by the following equation for *p*-polarized incident light can be obtained:

$$r_{p}=\frac{\left(M_{11}+M_{12}q_{N}\right)q_{1}-\left(M_{21}+M_{22}q_{N}\right)}{\left(M_{11}+M_{12}q_{N}\right)q_{1}+\left(M_{21}+M_{22}q_{N}\right)}.$$ (6)

The reflectivity, *R*_*p*_, of the combined multilayer structure is specified as shown in the following equation:

$$R_{p}={\left|r_{p}\right|}^{2}.$$ (7)

The performance characterization of an SPR-based sensor is basically contingent on the sensitivity, signal to noise ratio (SNR), and quality factor of the sensor. In order to obtain a better performance from an SPR-based biosensor, all the parameter’s higher values are preferred, except the full width at half maxima (FWHM) (Verma *et al.*, 2015a). The detection accuracy or the SNR and the quality factor achieve a higher value with a lower value of the FWHM (Maurya and Prajapati, 2016). The SPR angle and reflectance attributes change with the change in the RI of the sample analyte. As a fundamental performance indicating parameter, sensitivity is defined as the ratio of SPR angle change to the RI change of the sample analyte [Eq. (8)]. The RI change of the target analyte leads to a change in the SPR angle, and this parameter is calculated with the unit of deg/RIU (Maharana *et al.*, 2015; Choi *et al.*, 2011),

$$S=\frac{\Delta \theta_{SPR}}{\Delta n_{a}}.$$ (8)

Another crucial parameter for SPR-based biosensor is the detection accuracy (DA), which is also known as the SNR, and this can be determined using Eq. (9) from the SPR curve (Homola, 2008). In addition, detection accuracy or the SNR is a unitless parameter,

$$DA=SNR=\frac{\Delta \theta_{SPR}}{\Delta \theta_{0.5}}.$$ (9)

The FWHM (Δ*θ*_0.5_), or spectral width, is outlined as the span of the SPR curve for 50% of maximum intensity of reflected light. The parameter that is carrying a lion’s share of significance is the quality factor (QF) that depends both on Δ*θ*_0.5_ and the sensitivity, and it can be represented as (Homola, 2008)

$$QF=\frac{\Delta \theta_{SPR}}{\Delta n_{S}\Delta \theta_{0.5}};\;RIU^{-1}.$$ (10)

Notably, the detection accuracy, sensitivity, and quality factor as well as the decreasing value of the FWHM signify an SPR-based biosensor as better (Maurya and Prajapati, 2016). For the proposed sensor, all the simulations are conducted in MATLAB.

## IV. RESULTS AND DISCUSSION

**FIG. 2.**
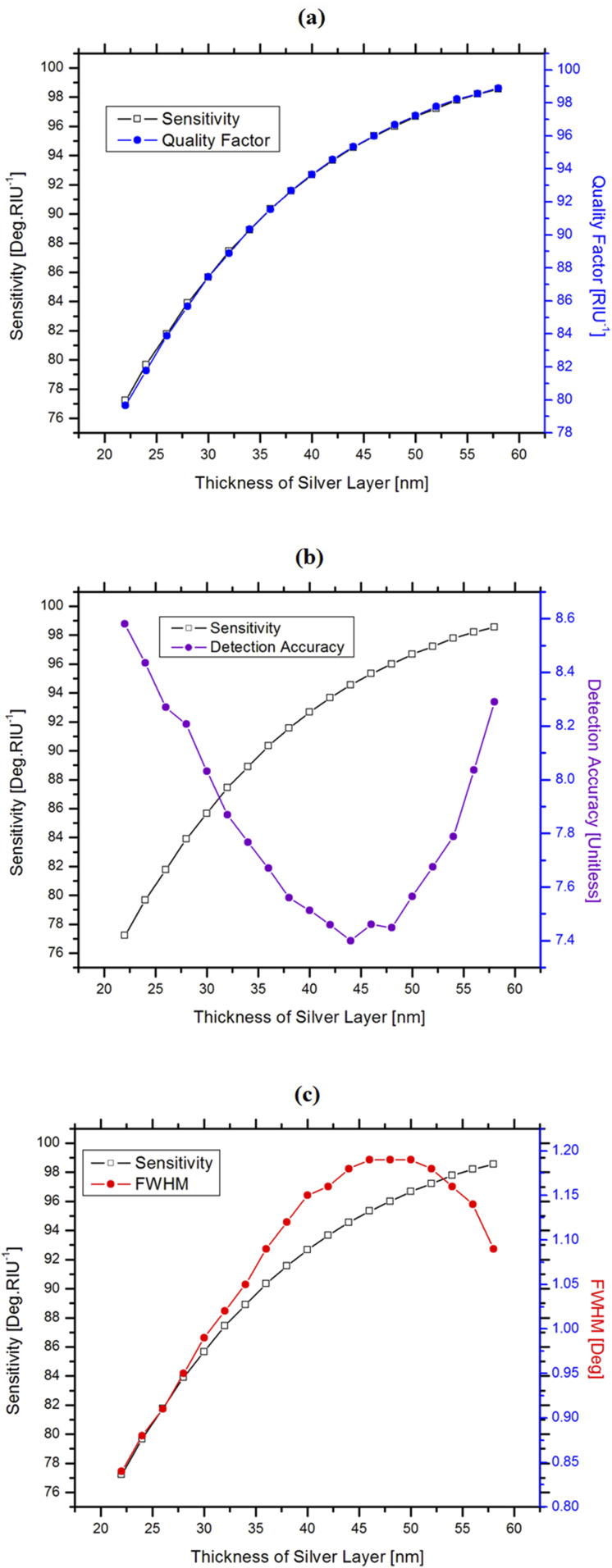
Variation in sensitivity and (a) quality factor, (b) detection accuracy, and (c) FWHM (full width at half maxima) with respect to the silver layer thickness.

A laser of 633 nm wavelength was chosen as the light source for the sensor. The initial RI of the sensing medium is 1.33 RIU, and the increment Δn_s_ in RI is considered to be 0.05 RIU. In addition, the initial thickness of the TiO_2_ layer is set to 5 nm. To optimize the thickness of the silver layer, we set its initial thickness at 22 nm and took the values of performance parameters and FWHM by varying the thickness up to 58 nm at an incremental rate of 2 nm. Figure 2(a) shows the variation in sensitivity and the quality factor, with a shift in Ag layer thickness. This curve shows an increasing trend in sensitivity and the quality factor with the increase in silver thickness.

The variation in sensitivity and detection accuracy is plotted in Fig. 2(b) with the variation in Ag layer thickness. Although the sensitivity increases with the increase in Ag layer thickness, the detection accuracy decreases initially up to a thickness of 44 nm, and then, it starts to increase again.

In addition, the sensitivity is potted against FWHM in Fig. 2(c) with respect to the thickness of the silver layer. The sensitivity increases with the increase in the thickness of the silver layer. Similarly, the FWHM increases with the increase in the silver layer’s thickness up to 46 nm, and then, it starts to decrease gradually. Figure 2(c) shows that these two lines cross at about 54 nm silver layer thickness. Previous studies found that at a thickness of 55 nm, silver showed a high quality factor with a lower FWHM, compared with the gold film for SPR-based sensor applications (Mukhtar *et al.*, 2018). However, in this study, 58 nm is found to be the best thickness for silver with respect to the sensitivity, quality factor, and detection accuracy of the proposed sensor. Beyond this thickness, the quality factor and detection accuracy were found to be unchanged. Thus, the thickness of the silver film was chosen as 58 nm for this proposed sensor structure, along with different material layers. In addition, silver in a multi-layered structure was found to have an enhanced quality factor compared to the single layer (Tran *et al.*, 2017).

**FIG. 3.**
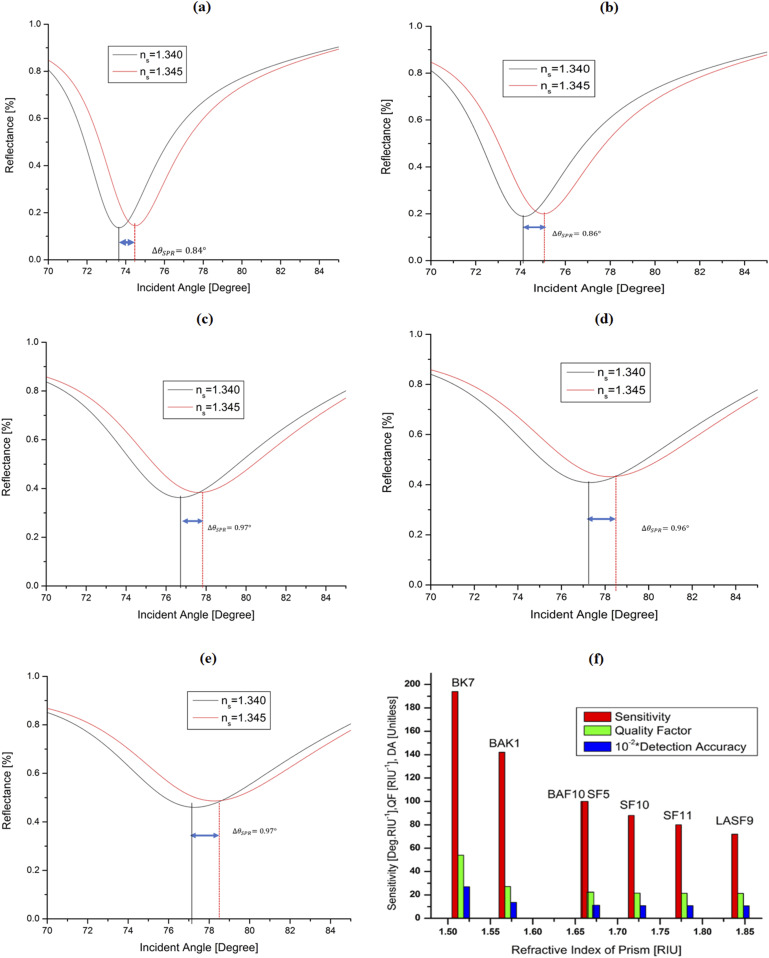
Variation in reflectance for four different sensor structures: (a) silver; Δ*θ*_*SPR*_ = 0.84°, (b) silver–graphene; Δ*θ*_*SPR*_ = 0.86°, (c) silver–MoSe_2_; Δ*θ*_*SPR*_ = 0.97°, (d) silver–MoSe_2_–graphene; Δ*θ*_*SPR*_ = 0.96°, and (e) TiO_2_–silver–MoSe_2_–graphene; Δ*θ*_*SPR*_ = 0.97°; (f) performance of different glass prisms.

The performance of the proposed sensor was compared with different layers of materials to check the sensor’s overall performance. The refractive index of the sensing medium is given as *n*_*a*_ = *n*_*s*_ + Δ*n*_*s*_, where Δ*n*_*s*_ represents the variation in the RI of the target solution medium. As shown in Fig. 3, the reflectance of five SPR sensor structures is presented with the variation in the incident angle, where the structures are (a) Ag, (b) Ag–graphene, (c) Ag–MoSe_2_, (d) Ag–MoSe_2_–graphene, and (e) TiO_2_–Ag–MoSe_2_–graphene. In this computation, the change in the RI of the sensing solution is considered as Δn_s_ = 0.005 RIU. As shown in Fig. 3(e), the shift in the dip in reflectance spectra is the highest after the introduction of the TiO_2_ layer with the Ag–MoSe_2_–graphene hybrid structure, which is 0.97°. In contrast, the lowest shift (0.84°) is observed for the Ag layer only [see Fig. 3(a)]. In this proposed sensor structure, the graphene layer was used due to its “significant properties such as strong adsorption of molecules, signal amplification by optical, high carrier mobility, electronic bridging, ease of fabrication and therefore, have established as an important sensitivity enhancement substrate for SPR” (Patil *et al.*, 2019; Hassan and Khan, 2014). Due to these characteristics, many recent studies also used the graphene layer for sensor applications (Bijalwan *et al.*, 2020; Moznuzzaman *et al.*, 2020; and Moznuzzaman *et al.*, 2021).

The selected multilayered structure, that is, a TiO_2_ layer, a thin silver film, a single sheet of MoSe_2_, and a single graphene sheet, can be implemented by the method of deposit and transfer on a prism. In terms of prism selection, sensitivity, quality factor, and detection accuracy were taken into account, and the results are illustrated in Fig. 3(f) as a function of the RI of the prism. The highest sensitivity, quality factor, and detection accuracy were found at an RI of 1.5151, and it is a BK7 prism. Thus, the BK7 prism was chosen for this proposed sensor. Due to these characteristics of the BK7 prism, it was used in many previous studies (Cherifi and Bouhafs, 2017; Tran *et al.*, 2017; and Prabowo *et al.*, 2017).

Table II lists the main performance parameters such as sensitivity, quality factor, and detection accuracy of the SPR sensor with different composite structures. It was revealed that the sensor with TiO_2_–Ag–MoSe_2_–graphene multilayers achieved the highest sensitivity. It was found that the proposed sensor’s sensitivity (194°/RIU), quality factor (54.0390 RIU^−1^), and detection accuracy (0.2702) outperformed those of other single and multi-layered structures, except the FWHM. Therefore, a TiO_2_–Ag–MoSe_2_–graphene multilayer structure was chosen for the proposed sensor.

**TABLE II.** Performance comparison of different SPR-based sensor structures. Boldface denotes the proposed SPR sensor structure.

Sensor structure	FWHM (deg)	Sensitivity (deg RIU^−1^)	Quality factor (RIU^−1^)	Detection accuracy (unitless)
Ag	5.26	168	31.9391	0.1597
Ag–graphene	5.60	172	30.7142	0.1536
Ag–MoSe_2_	6.10	194	31.8032	0.1590
Ag–MoSe_2_–graphene	5.53	192	34.7197	0.1736
**TiO**_**2**_**–Ag–MoSe**_**2**_**–graphene**	**3.59**	**194**	**54.0390**	**0.2702**

### A. Detection of SARS-CoV-2

There are two distinctive roles of antibodies: one is to fix exactly to their specific antigens and the other is to prompt an immune reaction against the engaged antigen by employing other cells and molecules (Sela-Culang *et al.*, 2013). The pair between an antibody and an antigen includes innumerable non-covalent contacts between the epitope and the paratope (Sela-Culang *et al.*, 2013). Proper immobilization of antibodies as biorecognition elements on the sensor surface is a significant task in order to achieve better performance of the biosensor (Sahoo *et al.*, 2016). This immobilization of antibodies on the sensor surface is mostly accomplished by either the physical adsorption method (Wang *et al.*, 2010; Liedberg *et al.*, 1983), that is, a weak electrostatic bond, or by permanent covalent bonding through revealing functional groups of easily accessible amino acid. Direct and physical immobilization has an advantage: the antibody molecules are very adjacent to the sensing surface, and this is highly conducive for achieving higher sensitivity (Sahoo *et al.*, 2016). For the proposed sensor, we immobilized CR3022 antibody molecules for detecting SARS-CoV-2 antigens (Yuan *et al.*, 2020). The CR3022 is a “Recombinant monoclonal antibody to COVID-19 & SARS-CoV S-glycoprotein. Manufactured using AbAb’s recombinant platform with variable regions (i.e., specificity) from the B-cell clone CR3022” (Absolute Antibody, 2020). The SARS-CoV-2 spike protein (S-glycoprotein) promotes access into cells and is the key target of the CR3022 antibody (Haynes *et al.*, 2007). “This antibody binds the amino acids 318–510 in the S1 domain of the SARS-CoV-2 (COVID-19) spike protein” (Absolute Antibody, 2020). The immobilization of SARS-CoV-2 antigens on the CR3022 antibodies increases the RI of the sensing layer, and this increased RI leads to a right shift in the SPR angle. By investigating this angular shift using attenuated total reflection (ATR), the existence of SARS-CoV-2 in the target sample can be determined.

**FIG. 4.**
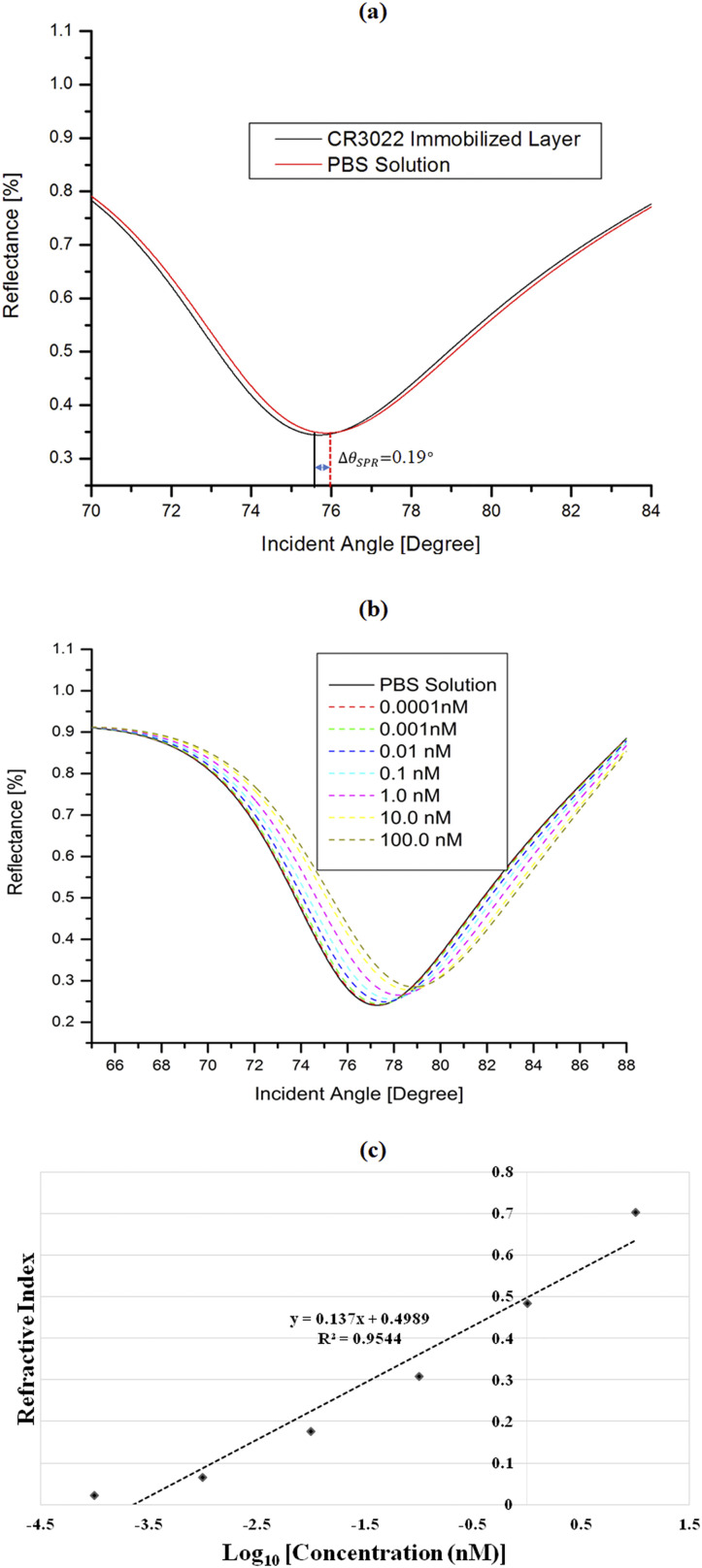
(a) Surface plasmon resonance curve for bare sensors and surfaces immobilized with the SARS-CoV-2 antibody (Δ*θ*_*SPR*_ = 0.19°), (b) SPR reflectivity curves for the graphene/SARS-CoV-2 antibody film in contact with various SARS-CoV-2 S-glycoprotein solution concentrations ranging from 0.0001 to 100 nM, and (c) change in the refractive index with respect to the change in the SARS-CoV-2 S-glycoprotein solution’s concentration for the proposed sensor.

Figure 4(a) shows the reflectance vs incident angle curves. The resonance angle of antibody coating on the graphene layer is 77.67°, whereas the resonance angle with the phosphate-buffered saline (PBS) solution is 77.86°.

In this technique, the collected SPR angle shift makes the conditions regarding either the presence of SARS-CoV-2 or not possible. The borderline criterion is signified by

$${\left(\Delta \theta_{SPR}^{Ab-Ag}\right)}_{\min}=\left|\theta_{SPR}^{Antibody}-\theta_{SPR}^{Antigen}\right|=0.19{}^{\circ},$$ (11)

where ${\left(\Delta \theta_{SPR}^{Ab-Ag}\right)}_{\min}$ directs the borderline value for the smallest change in the SPR angle. This value is for the PBS solution on the immobilization of antibody coating. As the immobilized CR3022 antibody only attaches to the antigen of SARS-CoV-2, the change in the SPR angle greater than this marginal value determines the existence of SARS-CoV-2. In this account, Eq. (12) (Diéguez *et al.*, 2012) reveals the increase in the RI for increases in the concentration of the SARS-CoV-2 sample solution,

$$n_{v}=n_{1}+c_{v}dn/dc,$$ (12)

where *c*_*v*_ is the concentration of the virus solution, *n*_1_ represents the RI of the immobilized CR3022 antibody, and the rate of change of RI with the change of concentration of SARS-CoV-2 solution is *dn*/*dc* = 0.181 cm^3^/gm (Diéguez *et al.*, 2012). As the RI of the sensing medium changes with the adsorption of the SARS-CoV-2 antigen, the SPR angle shifts rightward and makes a change in the propagation constant. Thus, this proposed improved performance SPR-based sensor is employed to identify SARS-CoV-2 using the angular investigation method.

A SARS-CoV-2 solution (range of concentration from 0.0001 to 100 nM) was inserted into the contact of the immobilized CR3022 film on the graphene surface. The SPR curve for SARS-CoV-2 solutions in contact with the specific graphene/CR3022 is shown in Fig. 4(b). The result shows that the SPR angle shifts insignificantly, i.e., 77.28° to 77.40°, when the target SARS-CoV-2 antigen concentration was varied from 0.0001 to 0.001 nM. This was perhaps because of the less amount of SARS-CoV-2 antigens in these lower concentrated solutions to fix onto the coated CR3022 antibody.

On the other hand, for high SARS-CoV-2 concentrations (0.01 and 10 nM), the SPR angles changed to a significant extent. The change was due to the increase in SARS-CoV-2 antigens that were being adsorbed on the antibody surface. Thus, it accordingly enlarged the change in the SPR angle. A similar type of finding was also reported in Fen *et al.* (2011).

The change in the SPR angle (*θ*_*SPR*_) was calculated by subtracting the SPR angle of the target solution from that of the reference PBS solution (77.28°). The change in the SPR angle is mainly dependent on the bindings of the CR3022 and SARS-CoV-2 antigens. More specifically, the greater the attachment of SARS-CoV-2 antigens to the CR3022 antibody surface, the greater the change in the SPR angle that can be observed. The shift in the SPR angle slightly increased due to the molar concentration increase in the SARS-CoV-2 antigen solution up to 0.001 nM. The variation in the SPR angle was observed in a range from 0.08° to 0.21° for the SARS-CoV-2 antigen concentration of 0.0001 to 0.001 nM. This outcome can be recognized by the additional immobilization between the target sample and the ligand, and this led to an increase in the RI of the detecting layer. This outcome is in line with Shankaran *et al.* (2005), who found that the increase in the SPR angle is the consequence of the developing bond of the antigen–antibody reaction. However, for the concentration of 100 nM, the SPR angle shift decreased compared to that of the previous concentrations. Due to the high concentration of the antigen, the antibody coated surface was entirely shielded and became congested (Omar *et al.*, 2018).

Finally, the modification of the RI due to the variation in different concentrations of the SARS-CoV-2 S-glycoprotein solution was also checked and is plotted in Fig. 4(c). This linear relationship (R^2^ = 0.9544) further justifies the value of the proposed sensor for SARS-CoV-2 detection in a highly sensitive manner as a similar nature of the relation was found for an SPR aptasensor to detect AIV H5N1 (Bai *et al.*, 2012).

The concentration of the SARS-CoV-2 S-glycoprotein solution was varied between 0.0000 and 10 nM, and related SPR angle shift is depicted in Table III. Analysis shows that a linear relationship exists between the concentration and SPR angle changes, which is a desired characteristic for a sensor (Omar *et al.*, 2018).

**TABLE III.** The SPR angle and related SPR angle shift for various concentrations of the SARS-CoV-2 S-glycoprotein solution (with related refractive index changes) in contact with the graphene/CR3022 antibody.

Concentration of SARS-CoV-2 S-glycoprotein solution (nM)	Refractive index of SARS-CoV-2 S-glycoprotein solution (RIU)	SPR angle, *θ*_*SPR*_ (deg)	SPR angle shift, Δ*θ*_*SPR*_ (deg)	Cumulative shift of the resonance angle, *C*Δ*θ*_*SPR*_ (deg)
0.0000	0.0000	77.28	0.00	0.00
0.0001	0.0220	77.32	0.04	0.04
0.0010	0.0660	77.40	0.08	0.12
0.0100	0.1760	77.61	0.21	0.29
0.1000	0.3080	77.87	0.26	0.51
1.0000	0.4840	78.21	0.34	0.85
10.000	0.7040	78.64	0.43	1.28

Similar linear trends were also found for the SPR angle shift and cumulative shift of the resonance angle against the SARS-CoV-2 S-glycoprotein solution’s concentration, as listed in Table III. This further ensures a positive characteristic of the proposed sensor (Omar *et al.*, 2020).

In summary, the proposed sensor’s sensitivity was found to be 13.40%, 11.34%, and 1.03% higher than the sensor structure with silver only, silver–graphene, and silver–MoSe_2_–graphene, respectively. In terms of the quality factor, the new sensor achieved about 40.9%, 43.16%, and 35.75% higher quality factor compared to the silver only, silver–graphene, and silver–MoSe_2_–graphene structures, respectively. Similarly, the detection accuracy was also found to be higher than that of the others.

## V. CONCLUSION

A theoretical design of a multi-layered surface plasmon resonance-based biosensor is proposed for virus detection. Compared with other available sensors, which are predominantly single-layered or optical fiber-based, this multilayered structure offers improved performance. For instance, the proposed sensor has a sensitivity of 194° RIU^−1^, a quality factor of 54.04 RIU^−1^, and a detection accuracy of about 0.2702. Therefore, this proposed sensor would offer highly sensitive detection of SARS-CoV-2. Although we used the CR3022 antibody for SARS-CoV-2, limitations exist with this antibody, as reported recently (Yuan *et al.*, 2020). However, the proposed sensor could be optimized for any new antibody. Most importantly, this type of sensor design could assist in detecting many different viruses, including SARS-CoV-2.

## Data Availability

The data that support the findings of this study are available from the corresponding author upon reasonable request.
